# Insecticide Residues in Cotton, Sorghum and Fallow Soil from the Nuba Mountains Cotton Corporation of South Kordofan State, Sudan

**DOI:** 10.5696/2156-9614-11.30.210608

**Published:** 2021-06-17

**Authors:** Amna Osman Mohamed, Azhari Omer Abdelbagi, Abdalla Mohammed Abdalla, Abd Elaziz Sulieman Ahmed Ishag, Ahmed Mohammed Ali Hammad, Nasradeen Adam Hamed Gadallah, Jang-Hyun Hur

**Affiliations:** 1 Department of Crop Protection, University of Kordofan, Sudan; 2 Department of Crop Protection, University of Khartoum, Sudan; 3 Department of Forest Conservation and Protection, University of Khartoum, Sudan; 4 Department of Biological Environment, Kangwon National University, The Republic of Korea

**Keywords:** insecticides, residues, cotton, sorghum, soil, Sudan

## Abstract

**Background.:**

Soil is the final depot of most environmental contaminants, including pesticides. Soil may be contaminated by pesticides as a result of direct application or drift during spray activities. Soil contamination with pesticide residues may affect the quality of food crops, animal products, and soil micro-organisms which may in turn negatively affect human health and the environment.

**Objectives.:**

The main objective of the current study was to determine the soil residues of commonly used pesticides in rain-fed crops grown by the Nuba Mountains Cotton Corporation (NMCC) in South Kordofan state of Western Sudan.

**Methods.:**

Four locations (representing the four directions around the state capital Kadugli) were chosen for sample collection: Alefain (East Kadugli), Elmashaish (West Kadugli), Ed Dalling uncultivated area (North Kadugli) and Lagawa (Southwest Kadugli). Nine soil samples were randomly taken from each location representing areas under cotton, sorghum, and uncultivated land covered with natural vegetation. Soil samples were analyzed by gas chromatography (GC) equipped with electron capture detector (ECD) and GC-mass spectrometry (MS).

**Results.:**

The results generally indicated that organophosphate levels were greater than organochlorine and pyrethroids with heptachlor, malathion, and dimethoate present in all samples analyzed, while the level of p,p-dichlorodiphenyltrichloroethane (DDT) was below the detection limit. Endosulfan α and β isomers were detected in some samples. Dimethoate had the highest level (22.02 mg/kg), while β endosulfan was found at the lowest level (0.015 mg/kg). Generally, samples collected from cotton soils showed higher residue levels compared to sorghum soil with average concentrations of 307.25 mg/kg versus 58.63 mg/kg, respectively. Almashaish showed the highest residues levels followed by Alefain, Lagawa, and Ed Dalling with total residues of 57.56 mg/kg, 26.34 mg/kg, 22.63 mg/kg, and 17.07 mg/kg, respectively.

**Conclusions.:**

The current study sheds light on the residue levels of some of the commonly used pesticides in the cotton rain-fed scheme in South Kordofan State, western Sudan. The study calls for regular residue monitoring in various environmental components in the area and suggests possible management measures.

**Competing Interests.:**

The authors declare no competing financial interests.

## Introduction

Sudan has one the highest rates of pesticide use in the Middle East and Africa.^1^ The annual import of pesticides reached about 5000 tons for crop protection and control of vector borne diseases in 1990.^1^

However, this amount decreased to a range of 2000–3000 tons after the 1990's^2^–^3^ due to adoption of integrated pest management (IPM) programs, changes in agricultural policy, and reduction in areas allotted to cotton. Irrigated cotton growing areas were known for intensive use of pesticides.^2^,^4^–^5^ On the other hand, rain-fed cotton was known to receive relatively lower levels of pesticides (less than 10%) and attract less investigation.^2^ Among the most important rain-fed cotton growing areas in Sudan is the Nuba Mountains Cotton Corporation (NMCC) of South Kordofan State, Western Sudan. South Kordofan State is rich in crop diversity and famous for production of cereals, oil seeds, vegetables, fruits, and cotton.^6^ From 1986–2003 this area experienced intensive and extensive spraying of different groups of insecticides due to an increase in the population density of many agricultural and public health pests.^6^ Cotton and sorghum were the dominant crops grown with a three year crop rotation (cotton, sorghum, and fallow). Cotton is the main cash crop and is repeatedly sprayed with insecticides during the growing season, while sorghum and fallow soils do not receive authorized pesticide application. However, they may become contaminated from previous season applications, unauthorized use, or from nearby control operations. Earlier studies reported a measurable level of organochlorine insecticides in soil and blood samples from the surrounding areas in western Sudan.^7^

Monitoring the level of contamination with pesticide residues in soil is important for a safe environment and healthy production of food crops. Soil is the final depot of most applied pesticides. Studies have shown that only 0.1% of the applied pesticides reach the target pest^8^–^9^ and the remaining 99.99% of the applied dose goes to the environment and ultimately ends up in soil,^8^,^10^ thus presenting a potential source of contamination to crops, surface and underground water, living biota and the associated food chain.^8^ Although most pesticides degrade fairly quickly on crops and in soil, some may persist in one form or another for a longer time. Pesticide active ingredients can be inactivated in soil by adsorption to clay and organic matter or degraded by microorganisms that digest and decompose organic matter in soil. The ability of pesticides to be degraded by micro-organisms (biodegradability) or by chemical action varies according to their chemical structure, degree of exposure, types of microorganisms, and environmental factors such as soil temperature, moisture content and climatic conditions.^11^–^17^

**Abbreviations**
*NMCC*: Nuba Mountains Cotton Corporation
*CAS*: Chemical Abstracts Service Registry

Considering the past and current intensive use of pesticides in the NMCC area and the expected acute and/or chronic adverse effects on humans, grazing animals, and other non-target organisms together with the expected negative impact on the quality of food crops grown in the area, this study aimed to examine residue levels of some of the most commonly used pesticides in soil grown with cotton, sorghum and fallow soil in the NMCC.^6^ As the primary cash crop, cotton receives repeated spraying with many insecticides during the growing season, while sorghum and fallow soils do not receive authorized pesticide application, however they may become contaminated from previous season applications. They were included in the investigation because they may reflect residues from previous season use and/or unauthorized use or from nearby control operations. The absence of previous studies in this area further strengthens these goals. The specific objectives of the study were to determine the residue concentrations of some of the commonly used pesticides in soil grown with cotton, sorghum, and fallow land in the NMCC area, to determine the level of pesticide residues in the soil of various production areas in the state (east, west, north, and south), and to provide policy makers and officials with base line data needed for designing suitable mitigation measures and formulating future environmental management plans.

## Methods

Analytical standard (purity 99%) of heptachlor (1, 4, 5, 6, 7, 8-8-exo 1, 4-endo-5, 8 dimethano heptachloro13a, 4, 7a tetrahydro-4, 7-methanoindene) Chemical Abstracts Service Registry (CAS) No. 76-44-8, DDT (1, 1, 1-trichloro-2, 2 bis (p-chlorophenolethanoindene) CAS No. 50-29-3, malathion diethyl (dimethoxythiophosphorythio) succinate; S-1, 2-bis (ethoxycarbonyl) ethyl O, O-dimethyl phosphorodithioate CAS No. 121-75-5, endosulfan (1, 4, 5, 6, 7, 7-hexachloro-8, 9, 10-trinorbon-5-en-2, 3-ylenebisme-thylene) sulfite; 6, 7, 8, 9, 10, 10-hexachloro-1, 5, 5a, 6, 9, 9, 9a-hexahydr0-6, 9-methano-2, 4, 3-benz0-dioxathathipine3-oxide CAS No. 115-29-7; dimeton 5, 5, dimethyl-3-oxocyclohex-1-enyl dimethyl carbamates CAS No. 60-51-5, deltamethrin ([1*R*-[1α(S*),3α]]-cyano(3-henoxyphenyl) methyl 3-(2,2-dibromoethenyl)-2,2-dimethylc yclopropanecarboxylate) CAS No. [52918-63-5]; [52820-00-5], and dimethoate (*O,O*-dimethyl *S*-[2-(methylamino)-2-oxoethyl] phosphorodithioate) CAS No. 60-51-5 were provided by the National Chemical Laboratory, Ministry of Health, Khartoum. The manufacture of the analytical standard is Sigma-Aldrich Company. Gas chromatograpohy grade (99.98% pure) acetone, *n*-hexane, sodium chloride and anhydrous sodium sulphate were supplied by Cculab Scientific Co. Ltd, Khartoum, Sudan.

### Study area

The study area is located in South Kordofan State, between longitude 29°–32° east and latitude 9°–12° north. The area is characterized by semi-arid conditions and summer rains. The average maximum temperature ranges between 36 to 40°C, while the minimum temperature ranges between 17- 20°C. Rainfall ranges from 600 to 850 mm per year. Half of the area is covered by dark cracking clay soil and grown with sorghum, cotton, and sesame as well as grazing plants. A considerable area of the northern part of the state is covered by sandy soil cultivated with millet.^18^

### Interview

Agricultural inspectors were interviewed based on their knowledge of pests and pesticides used in the area. Short questions were asked to ten plant protection inspectors in South Kordufan state. Questions asked included years of previous experience, types of crops grown, major pest, types of pesticides applied, and number of spraying per growing season *(Supplemental Material Table 1–5).*

### Soil sampling and sample preparation

A total of 63 samples were taken from 4 locations representing soils under commonly grown crops (cotton and sorghum) and uncultivated land. Nine samples were taken from soil under each crop (sorghum, cotton, and fallow) in each location. Selected locations include Alefain, East Kadugli; Elmashaish, West Kadugli; Lagawa, Southwest Kadugli, and Ed Dalling,an uncultivated area North Kadugli (*Figure 1*). The selected sites are managed by the NMCC. The sample size, as well as the sample collection method, was carried out according to the grid pattern method (3×3) with nine total sample proportions. Each sample site represents one portion of the total sample. Two soil plugs were taken from each site. The two soil plugs combined to form the sample portion of that sample site.^19^

**Figure 1:**
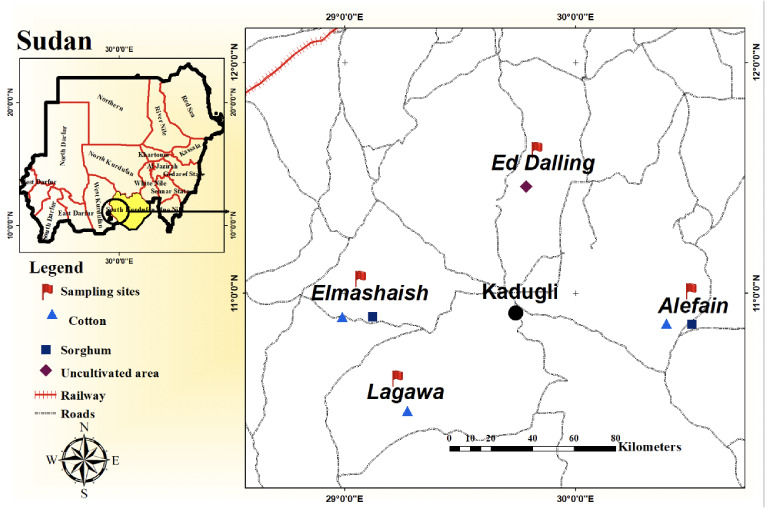
Sampling sites

**Figure 1:**
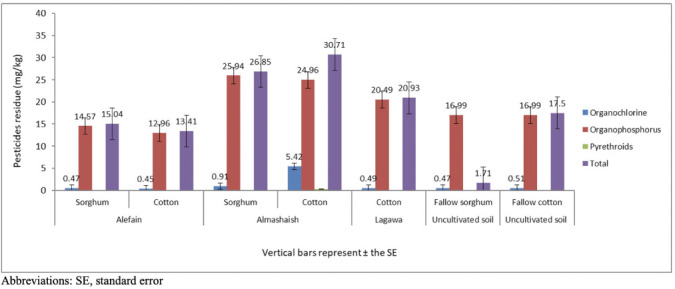
Total soil residue levels (mg/kg) of the main insecticide groups detected across locations

Before taking any soil samples, plant debris was removed from the soil surface. A soil auger of 17 cm length and 5 cm diameter was used to collect the samples. Four sub-samples were taken from different places and mixed thoroughly to make a composite sample (1 kg each). All samples were collected from the top 10 cm. Collected samples were kept separately in polyethylene bags, labeled with date and sample codes, and immediately transferred to the pesticide analytical laboratory of the Faculty of Agriculture, University of Khartoum. During sample collection, agricultural inspectors in the area were interviewed about pesticides used and their use pattern. Samples were left overnight to dry in the open air at room temperature. Big clods were broken by hand to a uniform reasonable size, crushed using pestle and mortar, and sieved through a 2-mm diameter sieve before extraction.

### Extraction and cleanup of samples

The Association of Official Analytical Chemists (AOAC)^20^ method No. 5 was followed for extraction and cleanup of samples. Fifty (50) grams of dried soil of each sample was placed into a 250-ml glass bottle and wetted with 15 ml of distilled water, and then 50 ml of re-distilled acetone and 50 ml of *n*-hexane were added. The bottle was tightly closed and placed firmly in an end-over-end shaker for three hours. The samples were then left to stand for 5 minutes to allow soil particles to settle down and then filtered through 18-mm filter paper. The filtrate was collected in a round bottle flask. This was placed in a rotary evaporator (Büchi Rotavapor R-200) at 40°C to reduce the filtrate volume to about 80 ml. The content was then transferred to a separatory funnel (capacity 1000 ml) then 600 ml distilled water and 10 ml of saturated sodium chloride solution (23% w/v) were added and shaken for one minute (the cock was opened several times to release pressure). The organic phase was transferred to a 500-ml separatory funnel, while the aqueous phase was re-extracted twice with 30 ml *n*-hexane. The combined *n*-hexane extracts were washed twice with 100 ml of distilled water and 5 ml of saturated sodium chloride solution. The organic phase was dried in a rotary evaporator to reduce the volume to 10 ml then filtered through filter paper containing 10 g of anhydrous sodium sulphate to absorb the moisture. The dried filtrate was then collected in tubes (10 ml) with a Teflon-lined screw cap, tightly closed, sealed with Teflon tape, and stored in a refrigerator at 4°C for GC analysis.

### Recovery test

Spiked samples were prepared and analyzed under similar conditions to check for method recovery. Sixteen grams of soil were weighted, divided into four groups of 4 g each. Each soil was placed in a glass bottle as previously mentioned, and then treated with analytical standards of candidate insecticides: heptachlor, 0.0134 mg/kg; p,p-DDT, 0.045 mg/kg; deltamethrin, 0.0135 mg/kg; α-β endosulfan, 0.0124 mg/kg; and endosulfan sulfate, 0.0156 mg/kg. Fortified samples were subject to the same extraction and clean up procedures. The recoveries of the method are greater than 91% as given in Supplemental Material Table 6.

### Gas chromatography (GC) analysis

All extracts of the soil samples were analyzed by Shimadzu GC model 2010 Japan, equipped with a Ni^63^ electron capture detector (ECD) and capillary column DB-5, 30 m length, 0.25 mm i.d. (internal diameter), Part No. 122-5032 and serial No. us6537762H. The stationary phase was 5% phenyl methyl polysiloxane (J & W Scientific) The operating temperature conditions of the oven, detector and injection port were 240°C, 300°C, and 280°C, respectively. Nitrogen (99.999% pure) was used as carrier gas at a flow rate of 3.33 ml/min.

One (1) μl of the analytical standard mixture (99% pure) of the candidate pesticides (α and β endosulfan, endosulfan sulfate, malathion, p,p-DDT, heptachlor, deltamethrin, and dimethoate) were injected using a 10-μl syringe with a needle length of 5 cm (Hamilton-Bonaduz Schweiz). The standard was injected three times or until approximately two consecutive similar peaks at the same retention time were obtained, followed by injection of the sample extract (1 μ/l). Each sample was injected three times. The concentrations of candidate pesticides were estimated from the peak area and expressed as mg/kg. The limit of detection (LOD), retention times, and maximum residue limits of the Food and Agriculture Organization of the United Nations (FAO) and World Health Organization are given in Supplemental Material Table 7.

### Gas chromatography with mass spectroscopy

Three representative samples were reanalyzed using a Shimadzu GCMS Qp2010 system (Japan) with an AOC-5000 auto sampler. The gas chromatography was fitted with a Rtx5-Ms capillary column 30 m x 0.25 mm id, 0.25 μm film thicknesses from Restek (UK) analytical column. The stationary phase (0.25 mm thickness) was 5% phenyl, methylpolysiloxane. Helium (purity ≥ 99.999%) was used as a carrier gas at a flow rate of 1.83 ml/min. The split less injection temperature was 200°C. The oven temperature was programmed from an initial temperature of 35°C (held for three minutes) then increased at 5°C min^−1^ to 200°C and held for 14 minutes. The mass spectrometer was operated with an electron impact (E I) source in scan mode. The electron energy was 70 e V, and the interface temperature was maintained at 280°C. The solvent delay was set to 2 minutes.

The Wiley7.LIB, NIST147.LIB, and NIST27.LIB libraries were searched for reconfirmation of identity of compounds detected by the GC-ECD. Libraries were searched for similarity of molecular weight and fragmentation pattern.

### Statistical analysis

Collected data were tabulated and statistically analyzed using the MINITAP software statistical package. Descriptive statistics was used in terms of means and standard deviation, in addition to median and range.

## Results

Interviews with agricultural inspectors indicated that they relied on different types of insecticides for crop protection purposes, mostly organophosphates (OPs), some pyrethroids, and occasionally organochlorines (OCs) (when available). Chemical control decisions were based on regular surveys of pests on cotton, and based on farmers' observations for sorghum, although the latter is rarely sprayed. The most commonly used insecticides reported by the respondents are presented in Supplemental Material Tables 3–7.

### Levels of pesticide residues detected in soils under cotton

The results revealed the presence of detectable levels of at least four pesticides in each location (*Table 1*). The level of organophosphorus pesticides was higher than other groups (organochlorines and pyrethroids). The highest levels were found in the samples collected from West Kadugli (Almashaish, (average 57.56 mg/kg and range (ND-17.16) mg/kg) followed by East Kadugli (Alefain, average 28.44 mg/kg, and range (ND-12.70) mg/kg), Southwest Kadugli (Lagawa, average 20.981 mg/kg and range (ND-19.82) mg/kg) and the uncultivated fallow soil of Ed Dalling, North Kadugli (average 17.47 mg/kg and range (ND-16.54) mg/kg) (*Table 1 and Figure 1*). Generally, cotton soils showed higher levels compared to sorghum soils and uncultivated land in various locations (*Table 1 and Figure 1*). Gas chromatography analysis revealed the presence of detectable levels of heptachlor, malathion, and dimethoate, and the absence of detectable levels of *p,p-* DDT in all analyzed samples.

**Table 1:** Total Soil Residue Levels (mg/kg) of the Main Insecticide Groups Detected Across Locations

**Locations**	**Insecticide group**	**Insecticides**	**Mean**	**S.D ±**	**Median**	**Range**	**Sample tested Positive (%)**
Alefain	Organochlorines	Heptachlor p,p-DDT α endosulfan β endosulfan Endosulfan sulphate **Total**	0.447 ND ND ND ND **0.447**	0.002 ND ND ND ND	0.448 ND ND ND ND	(0.445–0.449) ND ND ND ND	100 ND 100 ND ND
	Pyrethroids	Deltamethrin	ND	ND	ND	ND	ND
	Organophosphates	Malathion Dimethoate	0.343 12.316	0.462 0.460	0.462 12.440	(0.299–1.169) (11.807–12.702)	100 100
		**Total**	**12.959**				
Almashaish	Organochlorines	Heptachlor p,p-DDT α endosulfan β endosulfan Endosulfan sulphate **Total**	1.560 ND 1.290 2.060 0.509 **5.419**	1.398 ND 0.154 0.237 0.627	1.560 ND 1.290 2.060 0.509	(0.570–2.550) ND (0.200–2.380) (0.380–3.730) (0.066–0.590)	100 ND 100 100 100
	Pyrethroids	Deltamethrin **Total**	0.330 **0.330**	0.477	0.330	(ND-0.770)	100
	Organophosphates	Malathion Dimethoate **Total**	8.910 16.050 **24.960**	5.17 1.630	8.910 16.050	(5.250–12.570) (14.850–17.160)	100 100
**Lagawa**	Organochlorines	Heptachlor p,p-DDT α endosulfan β endosulfan Endosulfan sulphate **Total OC residues**	0.448 ND 0.040 ND ND **0.488**	0.004 ND 0.00045 ND ND	0.446 ND 0.039 ND ND	(0.445–0.454) ND (0.036–0.045) ND ND	100 ND 100 ND ND
	Pyrethroids	Deltamethrin	ND	ND	ND	ND	ND
	Organophosphates	Malathion Dimethoate **Total**	0.903 19.590 **20.493**	0.445 0.220	0.701 19.570	(0.595–1.413) (19.380–19.820)	100 100
Uncultivated soil	Organochlorines	Heptachlor p,p-DDT α endosulfan β endosulfan Endosulfan sulphate	0.447 ND 0.041 0.021 ND	0.007 ND 0.0002 0.002 ND	0.447 ND 0.042 0.021 ND	(0.446–0.448) ND (0.039–0.042) (0.019–0.024) ND	100 ND 100 100 ND
	Pyrethroids	Deltamethrin **Total**	ND **0.509**	ND	ND	ND	ND
	Organophosphate	Malathion Dimethoate **Total**	0.823 16.170 **16.993**	0.551 0.425	0.643 16.250	(0.385–0.144) (15.700–16.540)	100 100

*Abbreviations: ND, not detected; SD, standard deviation.*

The highest level detected in all locations corresponded to dimethoate at an average concentration of 22.02 mg/kg and range of 21.45–22.68 mg/kg, while the lowest concentration detected varied according to location: malathion in East Kadugli (Alefain, 0.34 mg/kg); deltamethrin in West Kadugli (Almashaish, 0.33 mg/kg); α endosulfan in Southwest Kadugli (Lagawa, 0.040 mg/kg), and β endosulfan in the uncultivated fallow soil of Ed Dalling, North Kadugli (0.021 mg/kg).

### Levels detected in soil under sorghum

Sampling of sorghum soil was possible in two locations Alefain, East Kadugli and Almashaish, West Kadugli, in addition to uncultivated soil. There was no sorghum grown in the south of the state during that season. The soil of West Kadugli showed the highest level, followed by East Kadugli, and lastly the uncultivated soils. Generally, organophosphorus compounds were found at higher levels compared to organochlorines, while pyrethroids were below the detection limit (*Table 2 and Figure 1*). The analysis of soil samples at the Alefain site showed the presence of detectable levels of heptachlor, βendosulfan, malathion, and dimethoate. No detectable levels of p,p-DDT, α endosulfan, endosulfan sulfate, and deltamethrin were found. The highest level detected in Alefain corresponds to dimethoate at an average concentration of 13.75 mg/kg and a range of 11.86–15.42 mg/kg, while the lowest level detected corresponds to α endosulfan (average 0.015 mg/kg and range ND-0.015 mg/kg). The soil analysis of the Almashaish site West Kadugli showed the presence of heptachlor, α endosulfan, β endosulfan, malathion, and dimethoate in all samples analyzed, while the levels of p,p DDT, endosulfan sulfate, and deltamethrin were below the detection limit (*Table 2*). The highest levels detected at this site corresponded to dimethoate (average 22.02 mg/kg and range of 21.25–22.68 mg/kg), while the lowest level detected corresponded to β endosulfan (average 0.129 mg/kg and range of 0.099–164 mg/kg).

**Table 2:** Concentration (mg/kg) of Insecticide Residues Detected in Soil under Sorghum in South Kordofan State

**Locations**	**Groups of Insecticides**	**Insecticides**	**Mean**	**S.D ±**	**Median**	**Range**	**Sample tested positive (%)**
Alefain	Organochlorines	Heptachlor p,p- DDT α endosulfan β endosulfan Endosulfan sulphate	0.450 ND 0.015 ND ND	0.010 ND 0.001 ND ND	0.450 ND 0.015 ND ND	(0.450–0.460) ND (ND-0.015) ND ND	100 ND 100 ND ND
	Pyrethroids	Deltamethrin **Total**	ND **0.465**	ND	ND	ND	ND
	Organophosphates	Malathion Dimethoate **Total**	0.779 13.752 **14.573**	0.301 1.546	0.715 13.926	(0.505–1.333) (11.861–15.420)	100 100
Almashaish	Organochlorines	Heptachlor p,p-DDT α endosulfan β endosulfan Endosulfan sulphate	0.645 ND 0.139 0.129 ND	0.031 ND 0.035 0.327 ND	0.654 ND 0.150 0.125 ND	(0.610–0.670) ND (0.099–0.161) (0.099–0.164) ND	100 ND 100 100 ND
	Pyrethroids	Deltamethrin **Total**	ND **0.913**	ND	ND	ND	ND
	Organophosphates	Malathion Dimethoate **Total**	3.917 22.019 **25.936**	0.584 0.617	4.007 21.929	(3.330–4.410) (21.450–22.680)	100 100
Uncultivated soil	Organochlorines	Heptachlor	0.447	0.002	0.446	(0.445–0.450)	100
		p,p-DDT	ND	ND	ND	ND	ND
		α endosulfan	0.041	0.002	0.042	(0.039–0.042)	100
		β endosulfan	0.020	0.003	0.019	(0.018–0.024)	100
		Endosulfan sulphate	ND	ND	ND	ND	ND
	Pyrethroids	Deltamethrin **Total**	ND **0.468**	ND	ND	ND	ND
	Organophosphates	Malathion Dimethoate **Total**	0.823 16.170 **16.993**	0.551 0.425	0.643 16.250	(0.385–0.144) (15.700–16.540)	100 100

*Abbreviations: ND, not detected: SD, standard deviation.*

The analysis (*Table 2*) of soil samples from uncultivated soil indicated the presence of detectable levels of heptachlor, α endosulfan, β endosulfan, malathion, and dimethoate in all samples analyzed, while the levels of p,p-DDT, endosulfan sulfate, and deltamethrin were below the detection limit (*Table 2*). The highest levels detected correspond to dimethoate (average 16.17 and ranges of 1.57–16.54 mg/kg), while the lowest levels detected belonged to β endosulfan (average 0.02 and range 0.018–0.024) mg/kg).

## Discussion

The results generally indicated the presence of detectable residues of some insecticides in all soil samples analyzed. Heptachlor, malathion and dimethoate were detected in all samples, while the level of p,p-DDT was below the detection limit. On the other hand, endosulfan β, endosulfan sulfate, and deltamethrin were found in cotton soil at one location only (Almashaish). The detection of some compounds in these locations can be explained by their use in crop protection in this area as confirmed by interviews with agricultural inspectors in the area. Limited previous studies in the rain-fed areas of western Sudan reported the detection of measurable levels of organochlorine residues in soil and blood samples from north and south Kordofan State.^4^,^7^ The group with the highest level detected was organophosphates followed by organochlorine and pyrethroids. This may be explained by the ongoing use of OPs in the area as well as from nearby control operations, especially those conducted against pests such as desert locusts or others (birds, sorghum bug, (*Agonoscelis pubescens*)) (Plant Protection Directorate (PPD) Personal Communication 2021). Organochlorine pesticides were banned in Sudan in 2004.^21^ They were known for their long environmental persistence, bioaccumulation along the food chain, and in the fat bodies of higher animals.^22^–^24^ Therefore, the presence of their residues in soil even after being banned or restricted in Sudan is expected based on the previous history of their heavy use and long environmental persistence. The pyrethroid deltamethrin was detected at one location in cotton soil only and this can be explained by its recent use in the area. Deltamethrin is a pyrethroid insecticide which has short environmental persistence and can be easily lost from soil,^25^–^26^ therefore, the absence of its residues in most samples can be explained by its possible conversation by various environmental factors.^24^ Dimethoate and malathion were detected in all samples and the level of dimethoate was the highest (22.02 mg/kg). This may be explained by their continuous use for crop protection purposes especially against cotton pests as indicated by the interviews with agricultural inspectors in the area. Similarly, heptachlor was also detected in all samples analyzed and this agrees with the results of previous studies of soil, water and blood samples from adjacent and/or other areas of the Sudan.^2^,^5^,^7^,^27^ Endosulfan and its metabolite endosulfan sulfate were detected, although at the lowest level 0.015 mg/kg for α endosulfan, with endosulfan sulfate detected in one location of cotton soil, while not detected in sorghum soil. Endosulfan was used to constitute at least 50% of the annual spray regime in cotton and was permitted for use against cotton pests in the Sudan until 2016 (Plant Protection Directorate (PPD) Personal Communication 2021), while not permitted for use in sorghum. Therefore, the presence of its residues and/or its metabolite endosulfan sulfate is expected.^28^–^29^ The absence of p,p-DDT residues may be explained by the possible environmental conversions to p,p-DDE and p,p-DDD as found in previous studies.^26^,^30^–^33^

Generally, cotton soil showed higher residue levels compared to sorghum soil and this can be explained by the fact that cotton is a cash crop that relies heavily on pesticides for crop protection purposes due to its pest complexity and sensitivity to pest damage, while sorghum is grown by traditional methods and normally receive no pesticide application. Similar to other agricultural corporations, MNCC had a crop protection department which conducts regular survey of cotton pests and recommends suitable control operations (Plant Protection Directorate (PPD) Personal Communication 2021). In general, West Kadugli soils (Almashaish), showed higher residues level compared to East Kadugli (Alefain) and Lagawa. It is worth mentioning that farmers in West Kadugli (Almashaish) regularly grow cotton, while in other locations (Alefain and Lagawa) they stopped growing cotton in 1993. This may explain the decreased soil residue levels in these locations (Alefain and Lagawa). On the other hand, the level detected in the fallow soil of Ed Dalling was even lower than that of Lagawa and Alefain. This may be explained by the fact that Ed Dalling samples were taken from non-cultivated fallow soil. Despite the detectable levels of some OPs and OCs found in soil, their presence may be explained by possible contamination by windblown dust or air drift during the active spray season. This argument agrees with Cohen and Pinkerton^34^ who reported measurable levels of OPs and OCs in windblown dust in the United States. The movement of residues by various environmental factors was reported.^24^

### Study limitations

The present study focused on the soil residues of some of the most commonly used pesticides. Residue levels in food sources and other environmental compartments were not assessed. Since the current study reported measurable levels of some pesticides and the study was performed with a limited number of samples, this indicates the need for a regular monitoring program of residues of all pesticides in the crops grown in the area and the surrounding environment. The study did not address the level of awareness of pesticide safety among farmers, which should be covered in future work. Proposals of suitable management plans and possible mitigation measures are also needed.

## Conclusions

Heptachlor, malathion and dimethoate were detected in all samples analyzed in the present study. Dimethoate was found at the highest level (22.02 mg/kg), while malathion and heptachlor were found at lower levels (8.91 mg/kg and 1.56 mg/kg, respectively). Cotton soil showed higher residue levels compared to sorghum soil with average concentrations of 43.69 mg/kg versus 41.46 mg/kg, respectively. Almashaish in west Kadugli had the highest residues levels, followed by Alefain, Lagawa, and Ed Dalling with total residues of 57.56 mg/kg, 27.95 mg/kg, 20.58 mg/kg, and 17.07 mg/kg, respectively.

The current study sheds light on the residue levels of some of the commonly used pesticides in the cotton rain-fed scheme in South Kordofan State, western Sudan. The study calls for regular residue monitoring in various environmental components in the area and suggests possible management measures.
