# Supplementary material for: Insecticide Residues in Cotton, Sorghum and Fallow Soil from the Nuba Mountains Cotton Corporation of South Kordofan State, Sudan
Source: J Health Pollut. 2021 Jun 17;11(30):210608. doi: 10.5696/2156-9614-11.30.210608 (PMC8276731; doi:10.5696/2156-9614-11.30.210608)
Supplement: Supplementary file 1 [file Mohamed_Supplemental_Material.docx]

**Supplemental Material**

**Insecticide Residues in Cotton, Sorghum and Fallow Soil from the Nuba Mountains Cotton Corporation of South Kordofan State, Sudan**

**Table 1.** Most Commonly Used Pesticides Reported by Interviewed Crop Protection Inspectors in Study Area

| **Percentage** | **Number** | **Pesticides** |
| --- | --- | --- |
| 100 | 10 | Endosulfan |
| 30 | 3 | DDT |
| 30 | 3 | Heptachlor |
| 100 | 10 | Malathion |
| 100 | 10 | Deltamethrin |
| 80 | 8 | Dimethoate |

Abbreviation: DDT, dichlorodiphenyltrichloroethane

**Table 2:** Interviewer Length of Experience

| **Number of respondents** | **Experience years** |
| --- | --- |
| 2 | 4 |
| 2 | 6 |
| 4 | 12 |
| 2 | 14 |

**Table 3:** Number of Sprays per Growing Season

| Number of sprays per season | Number of respondents | Percentage |
| --- | --- | --- |
| 2 | 3 | 30 |
| 3 | 4 | 40 |
| 4 | 2 | 20 |
| 5 | 1 | 10 |

**Table 4:** Type of Crop Grown

| **Number of respondents** | **Crop type** |
| --- | --- |
| 10 | Cotton |
|  | Sorghum |

**Table 5:** Major Pests

| **Number of respondents** | **Pest** |
| --- | --- |
| 10 | African bollworm |
|  | Jassid |
|  | Stem borer |
|  | Weeds |
|  | Rodent |

# Table 6: Percentage Recovery of Candidate Insecticides from Fortified Samples

| **Insecticide types** | **Recovery (%)** |
| --- | --- |
| p,p- DDT | 100 |
| Heptachlor | 100 |
| α Endosulfan | 98 |
| β Endosulfan | 100 |
| Endosulfan sulfate | 99 |
| Deltamethrin | 91 |
| Malathion | 96 |
| Dimethoate | 98 |

Abbreviation: DDT, dichlorodiphenyltrichloroethane

# Table 7: Minimum Detection Limits (LOD), Retention Times and Maximum Residue Limits (MRLs) of the Studied Insecticides

| **Pesticides** | **Limit of detection (ppm)** | **Retention time (min.)** | **MRLs (ppm)* ^1-2^** |
| --- | --- | --- | --- |
| p.p-DDT | 0.150 | 24.96 | 0.001 |
| Heptachlor | 0.445 | 27.84 | 0.030 |
| Malathion | 0.333 | 30.70 | 0.030 |
| α Endosulfan | 0.015 | 34.54 | 0.02 |
| β Endosulfan | 0.018 | 35.03 |  |
| Endosulfan sulfate | 0.307 | 32.26 | - |
| Dimethoate | 0.149 | 39.46 | 0.050 |
| Deltamethrin | 0.063 | 43.68 | - |

“-“: Not available

Abbreviation: DDT, dichlorodiphenyltrichloroethane

*Sources; Food and Agriculture Organization of the United Nations (FAO) and World Health Organization; Lamia and Amani **^1-2^**

**Figure 1.** Chromatogram of standard insecticide mixture

**Figures 2-8. Excerpts of chromatograms of analyzed samples**

**Figure 2.** Typical chromatogram of cotton soil in Alefain area

**Figure 3.** Typical chromatogram of cotton soil in Almashaish area

**Figure 4.** Typical chromatogram of cotton soil in Lagawa area

**Figure 5.** Typical chromatogram of fallow soil (cotton) in Ed Dalling area

**Fig. 6** Typical chromatogram of sorghum soil in Alefain

**Figure 7.** Typical chromatogram of sorghum soil in Almashaish

**Figure 8.** Typical chromatogram of fallow soil (sorghum) in Ed Dalling area

1. Food and Agriculture Organization of the United Nations/World Health Organization. Codex Alimentarius commission additives and contaminants Joint FAO/WHO food standards programme. ALIWORM 01/12A. Pesticide Programme Residue Monitoring, pp. 1-289. (2004) Accessed [2021 March 30]. Available from: [www.fao.org](http://www.fao.org)
2. Lamia A. Albedair & Amani S. Alturiqi. Evaluation of pesticide residues in the irrigation water, soil and assessment of their health risks in vegetables from sub-urban areas around Riyadh district, Saudi Arabia, Environmental Forensics, (2020). https://doi.org/10.1080/15275922.2020.1805826
